# Liquid facets-Related (lqfR) Is Required for Egg Chamber Morphogenesis during *Drosophila* Oogenesis

**DOI:** 10.1371/journal.pone.0025466

**Published:** 2011-10-17

**Authors:** Peter A. Leventis, Tanya R. Da Sylva, Nimerta Rajwans, Sylwia Wasiak, Peter S. McPherson, Gabrielle L. Boulianne

**Affiliations:** 1 Program in Developmental and Stem Cell Biology, Hospital for Sick Children, Toronto, Ontario, Canada; 2 Department of Cell and Systems Biology, University of Toronto, Toronto, Ontario, Canada; 3 Montreal Neurological Institute, McGill University, Montreal, Quebec, Canada; 4 Department of Molecular Genetics, University of Toronto, Toronto, Ontario, Canada; Institut Pasteur, France

## Abstract

Clathrin interactor 1 [CLINT1] (also called enthoprotin/EpsinR) is an Epsin N-terminal homology (ENTH) domain-containing adaptor protein that functions in anterograde and retrograde clathrin-mediated trafficking between the trans-Golgi network and the endosome. Removal of both *Saccharomyces cerevisiae* homologs, Ent3p and Ent5p, result in yeast that are viable, but that display a cold-sensitive growth phenotype and mistrafficking of various vacuolar proteins. Similarly, either knock-down or overexpression of vertebrate CLINT1 in cell culture causes mistrafficking of proteins. Here, we have characterized *Drosophila CLINT1*, *liquid-facets Related* (*lqfR*). LqfR is ubiquitously expressed throughout development and is localized to the Golgi and endosome. Strong hypomorphic mutants generated by imprecise P-element excision exhibit extra macrochaetae, rough eyes and are female sterile. Although essentially no eggs are laid, the ovaries do contain late-stage egg chambers that exhibit abnormal morphology. Germline clones reveal that LqfR expression in the somatic follicle cells is sufficient to rescue the oogenesis defects. Clones of mutant *lqfR* follicle cells have a decreased cell size consistent with a downregulation of Akt1. We find that while total Akt1 levels are increased there is also a significant decrease in activated phosphorylated Akt1. Taken together, these results show that LqfR function is required to regulate follicle cell size and signaling during *Drosophila* oogenesis.

## Introduction

Vesicular trafficking is required in eukaryotes for the movement of membranes, proteins, and other bioactive molecules between cellular compartments. Examples include the transport of transmembrane receptors to the plasma membrane, exocytosis of neurotransmitters at the synapse, retrieval of synaptic vesicle membrane and protein machinery by endocytosis, and delivery of proteins and membrane for degradation to the lysosome. Although vesicle formation occurs by several processes, one of the best studied is clathrin-mediated vesicle formation.

Clathrin-mediated vesicle formation is found principally at three locations in the cell: at the plasma membrane during endocytosis; at the trans-Golgi network (TGN) during the formation of some vesicles that traffic to the endosome; and at the endosome for trafficking back to the TGN [1]. Adaptor proteins help mediate the recruitment of clathrin to appropriate membrane and sites of cargo enrichment. One such set of adaptors is the epsin family of proteins, which are found at the plasma membrane. They contain a phosphatidylinositol 4,5-bisphosphate-binding ENTH domain [2], and motifs for interaction with clathrin [3], other cargo adaptors, and ubiquitylated proteins. Epsins thus bind to a specific subset of membrane and to proteins marked for endocytosis by ubiquitin, and recruit clathrin and the tetrameric clathrin adaptor AP-2. The insertion of a helix of the ENTH domain between the polar heads of the lipid membrane is thought to then help induce curvature of the developing clathrin-coated pit [4].

At the TGN and endosome, the adaptor CLINT1 (also called EpsinR/enthoprotin in vertebrates and liquid facets-Related in *Drosophila*) has an analogous function in clathrin-coated vesicle formation and has a similar domain structure to epsins. The ENTH domain of vertebrate CLINT1 interacts with phosphatidylinositol 4-phosphate (PI4P) [5], [6], [7], which is enriched in TGN and endosome membranes. The C-terminal part of CLINT1 binds to clathrin, and to the γ-ear domain of the clathrin adaptor GGA and the γ-adaptin subunit of the heterotetrameric clathrin adaptor AP-1 [5], [7], [8], [9].

Disruption of CLINT1 function affects clathrin-mediated trafficking from the TGN to the endosome. Either RNAi-mediated knock-down or strong overexpression of CLINT1 in HeLa or COS cells disrupts the normal route of trafficking of the lysosomal protein cathepsin D resulting in secretion of a portion of the protein [5], [7]. Similarly, deletion of two *S. cerevisiae* proteins with CLINT function and mutants of the *Arabidopsis thaliana* homologue display defects in trafficking to the vacuole [10], [11], [12]. CLINT1 has also been implicated in retrograde transport from the endosome to the TGN [13].

Although its role in intracellular trafficking has been characterized in unicellular yeast and vertebrate cell culture, much remains unknown about the role of CLINT1 in normal development of a multicellular organism. Recently, studies in *Drosophila* have found that the CLINT1 homologue, *liquid facets-Related* (*lqfR*), is required for viability and, in the developing eye, *lqfR* is required for cell fate determination, cell proliferation and insulin-independent cell growth [14]. However, many questions on the developmental role of *lqfR* remain. To answer these questions we have independently characterized *lqfR*. We confirm that LqfR is ubiquitously expressed and is localized to the TGN and endosome. In addition, we have developed strong hypomorphic *lqfR* mutants that are viable, but are female sterile which allowed us to characterize the role of *lqfR* in oogenesis. Here we show that *lqfR* is required in the somatic follicle cells for normal egg chamber morphogenesis, and that reduction of *lqfR* disrupts Akt signaling in the follicle cells and affects the actin cytoskeleton in the oocyte.

## Materials and Methods

### Fly stocks

Flies were housed under standard conditions at room temperature. The following stocks were obtained from the Bloomington *Drosophila* Stock Center (Indiana University, Bloomington, IN): *l(3)03685*, *ry^506^/TM3*, *ry^RK^ Sb^1^ Ser^1^* (BL-11601), *w^1118^*; *Df(3R)Exel6191/TM6B*, *Tb^1^* (BL-7670), *UAS-Clc-EGFP* (BL-7107), *UAS-Rab4-mRFP* (BL-8505), *UAS-Rab11-GFP* (BL-8506), *FRT-82B P{w^+^*, *ry^+^}90E* (BL-2050), *FRT-82B N-myc* (BL-2034) and *FRT-82B Ubi-GFP(S65T)nls/TM6*, *Tb^1^* (BL-5628). Other stocks were from our laboratory except for *UAS-Rab5-GFP* (M. Gonzáles-Gaitán, University of Geneva, Geneva, Switzerland).

### Reagents

The CLINT1 EST GH02671 was obtained from Research Genetics/Invitrogen (Carlsbad, CA). Primary antibodies for immunofluorescence were obtained from the following sources: anti-syntaxin 16 (W. Trimble, Hospital for Sick Children, Toronto, ON), anti-p120 (Calbiochem), anti-spectrin (Developmental Studies Hybridoma Bank developed under the auspices of the NICHD and maintained by The University of Iowa, Department of Biology, Iowa City, IA) and anti-Hrs (H. Bellen, Baylor College of Medicine, Houston, TX). Primary antibodies for Western blot were: anti-Akt #9272 and anti-phospho-Ser^505^-*Drosophila* Akt1 #4054 (Cell Signaling Technology, Danvers, MA), anti-γ-Adaptin and anti-clathrin heavy chain (BD Transduction Laboratories), and anti-β-Tubulin, clone E7 (Developmental Studies Hybridoma Bank developed under the auspices of the NICHD and maintained by The University of Iowa, Department of Biology, Iowa City, IA). Secondary antibodies were obtained from Jackson Immunoresearch Laboratories (West Grove, PA) or Molecular Probes/Invitrogen. Unless otherwise noted, other chemicals and reagents were obtained from Sigma-Aldrich (St. Louis, MO).

### Generation of a hypomorphic *lqfR* Mutant

The line *l(3)03685* [15], which will be referred to as *lqfR^03685^*, contains a P-element insertion in the 5′ region of *lqfR* (full FlyBase name: *liquid facets-Related* FBgn0261279) and behaves as a hypomorph. To generate a deletion mutant line, the P-element was mobilized using standard genetic techniques. This led to the identification of the *lqfR* allele *lqfR^D66^*. Another line generated in the same screen, *lqfR^+A4^*, was a precise excision and was used as a genetic control in subsequent experiments.

### Northern blot analysis

Northern blots of embryonic, larval, and adult *Drosophila* mRNA (generously provided by S. Kim) were made and analysed as described [16].

### Generation of LqfR antibodies

Antibodies to a truncated protein lacking the ENTH domain were generated in two rats (3148-1 and 3148-2) and two rabbits (H7660 and H7666) by Antibodies, Inc. (Irving, CA) using recombinant (GST)-LqfR (residues 166–649) fused to a C-terminal 6XHis tag, generated using methods previously described [17]. Sera from all four animals recognized a major band at ∼90 kDa, and another at ∼200 kDa in immunoblots of wild-type animals. These bands were not detected by pre-immune serum and were absent in lysates prepared from *lqfR^D66^* homozygotes demonstrating that the antibodies were specific to LqfR. Additionally, rat 3148-2 and rabbit H7666 antisera did not detect any protein in *lqfR* null clones. Serum from rabbit H7666 was affinity purified with an S-tag fusion protein of residues 166–649 coupled to Sulfo-Link (Pierce, Rockford, IL) columns.

### GST pull-down assays

GST-fusions of full-length or C-terminal (aa 165–649) LqfR were produced using a pGEX-4T-1 vector containing a C-terminal 6xHis tag expressed in the *E. coli* strain BL-21 (Stratagene, La Jolla, CA). Adult rat brains were homogenized in Buffer A (10 mM HEPES-OH, pH 7.4, 0.83 mM benzamidine, 0.23 mM phenylmethylsulfonyl fluoride, 0.5 µg/ml aprotinin, and 0.5 µg/ml leupeptin) and a post-nuclear supernatant was obtained by centrifugation at 800 *g* for 5 minutes. The supernatant was incubated with 1% Triton X-100 +/− 150 mM NaCl for 30 minutes at 4°C and then centrifuged at 205,000 *g*. Aliquots of the Triton X-100-soluble lysate (2 mg of protein) were incubated for 2 hours or overnight at 4°C with GST fusion proteins pre-coupled to glutathione-Sepharose beads (Amersham/GE Healthcare, Piscataway, NJ). Following incubation, samples were washed three times in an appropriate buffer containing 1% Triton X-100, and specifically bound proteins were resolved by SDS-PAGE and processed for Western blot analysis.

### Immunohistochemistry

Ovaries were dissected in cold phosphate-buffered saline (PBS), pH 7.0, fixed for 15 minutes at RT in 5% paraformaldehyde in PBS then washed in PBS, and permeabilized with PBT (PBS with 0.3% Triton X-100). Ovaries were blocked in 5% normal secondary host serum and 0.2% bovine serum albumin (BSA) in PBT. Primary antibodies were diluted in blocking solution and used at the following concentrations: rat anti-LqfR 3148-2 (1∶500), affinity purified rabbit anti-LqfR H7666 (1∶100), rabbit anti-syntaxin 16 (1∶500), mouse anti-p120 (1∶100), and guinea pig anti-Hrs (1∶1,000). Secondary antibodies used were: donkey anti-rabbit-Cy3, donkey anti-rabbit-A488, donkey anti-rat Cy3, goat anti-guinea pig-A488, donkey anti-mouse-Cy3, and donkey anti-mouse-A488. Secondary antibodies were diluted 1∶500–1∶1000 in block or 1% BSA in PBT. For nuclear visualization ovaries were stained with the nuclear dye ToPro-3 (Molecular Probes/Invitrogen) According to manufacturer's directions. To visualize the actin cytoskeleton, ovaries were washed three times in PBS following secondary antibody staining, and incubated with 1 µM TRITC-phalloidin in PBS. Ovaries were mounted in antifade (2% DABCO (1,4-diazabicylo[2.2.2]octane), 70% glycerol, in 0.1 M Tris, pH 7.6). Confocal images were acquired on a Zeiss Axiovert 200 inverted microscope with a META emission scan head and LSM510 software. Epifluorescence images were acquired with a Leica DMRA-2 microscope equipped with a Hamamatsu Orca-ER digital camera.

### Immunoblotting

Whole animals or ovaries were lysed in 1× Laemmli sample buffer prepared with dithiothreitol. The equivalent of 0.1 animal (∼20 µg) or one ovary was loaded into each lane of a 10% acrylamide gel and proteins were resolved by SDS-PAGE. Following electrophoresis, proteins were transferred to PVDF membranes (Pall Life Sciences, Ann Arbor, MI) and membranes were blocked in 2% non-fat milk and 2% normal secondary host serum (Chemicon/Millipore, Danvers, MA). Primary antibodies were diluted in blocking solution as follows; crude rat anti-LqfR 3148-2 (1∶2,500), affinity purified rabbit anti-LqfR H7666 (1∶500), rabbit anti-Akt (1∶1,000), rabbit anti-phospho-Ser^505^-*Drosophila* Akt1 (1∶1,000), mouse anti-γ-Adaptin (1∶5,000), mouse anti-clathrin heavy chain (1∶1,000), and mouse anti-β-Tubulin (1∶1,000). Membranes were incubated with horseradish peroxidase-conjugated secondary antibody diluted in 1% BSA in TBST. The following secondary antibodies were used at 1∶10,000: donkey anti-rabbit-HRP, donkey anti-mouse-HRP, and goat anti-rat-HRP. Proteins were detected with Western Lightning chemiluminescent reagent (Perkin-Elmer, Waltham, MA).

### Germline and follicle cell mitotic clones

*FRT-82B lqfR^D66^* lines were generated by recombination of *lqfR^D66^* with *FRT-82B P{w^+^*, *ry^+^}90E* or *FRT-82B N-myc*. For germline clones, females of the genotype *w*; *pr pwn hsFLP/+*; *FRT-82B ^ovoD1–18^/FRT-82B lqfR^D66^* were heat shocked at 37°C for 60 minutes as second and early third instar larvae. For generating somatic clones, the females were also heat shocked at 37°C for 60 minutes for 2–4 times total on two consecutive days just prior to, or immediately following eclosion. Females were fed with yeasted food and ovaries were dissected 2–4 d later. For further analysis of somatic clones, *y w hsFL122/w* ; *FRT-82B N-myc lqfR^D66^/FRT-82B ubiGFP(S65T)nls* females were heat shocked as described above. Stages were determined as described by Spradling [18].

## Results

### *Drosophila lqfR* encodes two distinct transcripts

**Figure 1:**
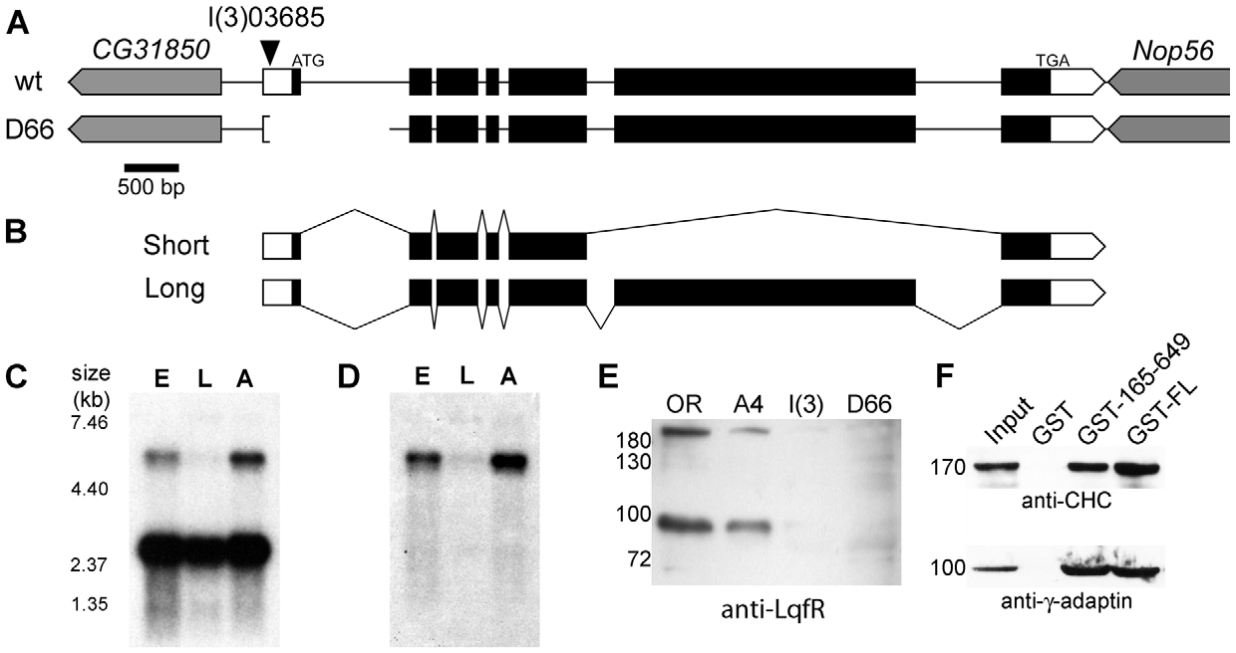
Characterization of *Drosophila* liquid facets-Related. (A) Genomic region of *lqfR*, based on FlyBase release FB2008_07. *lqfR* is on the right arm of chromosome 3 and is oriented 5′ to 3′ towards the telomere. It occupies 7.7 kb and is flanked at the 5′ end by CG31850 and on the 3′ end by Nop56, both which are on the opposite (-) strand. The ATG-Start and TGA-Stop codons for the small splice product are shown. Within *lqfR*, white boxes represent UTR and black boxes represent ORF. Gene orientation is indicated by the pointed bars. The location of the P element insertion in *lqfR* ^03685^ is shown by the arrowhead. The *lqfR* ^D66^ deletion is 1.1 kb. Scale bar, 500 bp. (B) The structure of the two predicted isoforms of *lqfR* is shown. Northern blots of embryonic, larval and adult mRNA probed with probes to *lqfR* exon 3–5 (C) and exon 6 (D). Marker sizes (in kb) are shown. (E) *lqfR* ^D66^ mutants have undetectable amounts of LqfR protein. Western blot of lysates from 1/10 homozygous wild-type Oregon R (OR), *lqfR* ^+A4^ (A4), *lqfR* ^03685^ (l(3)), or hypomorph *lqfR* ^D66^ (D66) early pupae detected with crude rat anti-LqfR 3148-2 antiserum. (F) GST-pull down assays. GST alone, or GST fusions of full-length LqfR (GST-FL) or amino acids 165–649 (GST-165–649) were incubated with a soluble rat brain extract. Specifically bound proteins were detected with antibodies against clathrin heavy chain (CHC) or γ-adaptin. An aliquot of 1/10 of the brain lysate input was processed in parallel. M_r_ of protein standards is shown in kDA (E, F).

To study the function of CLINT in a multicellular organism we first identified the *Drosophila melanogaster* homolog of CLINT1. By performing a TBlastN search [19] with human CLINT1 (gi∶40788895), we found a single candidate *Drosophila* gene (FlyBase name: *liquid facets-Related*; *lqfR*) consisting of 7 exons occupying 7.7 kb (Fig. 1A). Sequencing of an available EST, GH02671 (gi∶16767871), revealed that its 2.7 kb product, consisting of exons 1 through 5 plus exon 7 (equivalent to FlyBase transcript *lqfRC*, FBtr0299515), was predicted to be similar to that of vertebrate *CLINT*s.

To verify the size and number of *lqfR* transcripts, we probed a Northern blot with a probe made from EST GH02671. In addition to the expected 2.7 kb transcript seen in embryos, larvae and adults, there was also an ∼5 kb transcript expressed most highly in embryos and adults (Fig. 1B). Since the size of this band was smaller than the genomic region, the probe was not recognizing contaminating genomic DNA. To determine if this larger *lqfR* transcript contained exon 6, the predicted translation of which shows homology with the *S. cerevisiae* telomere-length regulating protein Tel2p [20], we reprobed the blot with an exon 6-specific probe (Fig. 1C). This probe detected only the ∼5 kb band seen in the previous Northern blot, demonstrating that exon 6 is present in an alternatively spliced product of *lqfR*. Although we were unable to produce and sequence a cDNA, the ∼5 kb transcript likely corresponds to FlyBase transcript *lqfR-RD* (FBtr0299516).

To determine the cellular and subcellular distribution of LqfR, we generated antibodies to a GST fusion of an ENTH domain-truncated protein (aa 165–649 of the product of the 2.7 kb transcript) in two rats and two rabbits. All four antisera recognized bands near 90 and 200 kDa in lysates prepared from wild-type flies (Fig. 1D and data not shown). These bands were reduced in lysate from the P-element containing allele *lqfR^03685^* and appeared to be absent in lysate from mutant *lqfR^D66^* (see below). The bands were also not recognized by pre-immune serum (data not shown) indicating that these bands represent LqfR proteins, although both are larger than the 69 kDa and 158 kDa expected sizes of the products of the 2.7 kb and 5 kb isoforms [20]. However, reduced mobility is consistent with what has been seen with other ENTH domain-containing proteins, including epsin and CLINT1 in vertebrates [9], [21] and EPSIN1 (which is functionally like CLINT1) in *Arabidopsis thaliana* [12].

### liquid facets-Related contains conserved protein domains

Based on the predicted sequence of the 2.7 kb transcript, the 649 aa *Drosophila* LqfR contains many of the protein motifs found in vertebrate and *S. cerevisiae* CLINTs. At the N-terminal, LqfR has an ENTH domain. By comparison with human CLINT1, the N-terminal homology extends from aa 8–172 (Fig. S1), which is slightly larger than the canonical ∼145 aa ENTH domain. Within this 165 aa region, *Drosophila* LqfR is very similar to that from human, with identical (130/165) or conserved residues (19/165) at over 90% of positions. Residues that are predicted to be important in phosphoinositide binding are conserved with those in vertebrate CLINT1 [6], [7], but not yeast Ent3p [22] or Ent5p [11], suggesting that LqfR may bind PI4P, and not PI(3,5)P_2_.

Although LqfR does not contain the type 2 clathrin boxes found in vertebrate homologs, there are two DLL tripeptide sequences (beginning at positions 352 and 477) and three similar DLF sequences (positions 356, 459, and 646) that may confer lower affinity clathrin binding than do the clathrin boxes [22], [23]. There are also two γ-adaptin ear interaction motifs [7], [24] beginning at positions 395 and 427 (the 1415 aa product of the 5 kb isoform is predicted to share the first 492 aa with the 649 aa product). To determine if LqfR can bind clathrin and γ-adaptin, we performed GST pull-down assays using recombinant full length (GST-LqfR^1–649^) or ENTH domain-truncated (GST-LqfR^165–649^)liquid facets-Related. Because of the lack of antibodies to the *Drosophila* clathrin and γ-adaptin proteins, we used lysates from rat brains. Both GST-LqfR^1–649^ and GST-LqfR^165–649^ were able to pull down clathrin and γ-adaptin (Fig. 1E). The same constructs could bind to in vitro translated *Drosophila* clathrin heavy chain and GGA (N.R., unpublished data). Thus, LqfR can interact with proteins important in TGN-endosome trafficking.

### liquid facets-Related is expressed in all tissues including the ovary

**Figure 2:**
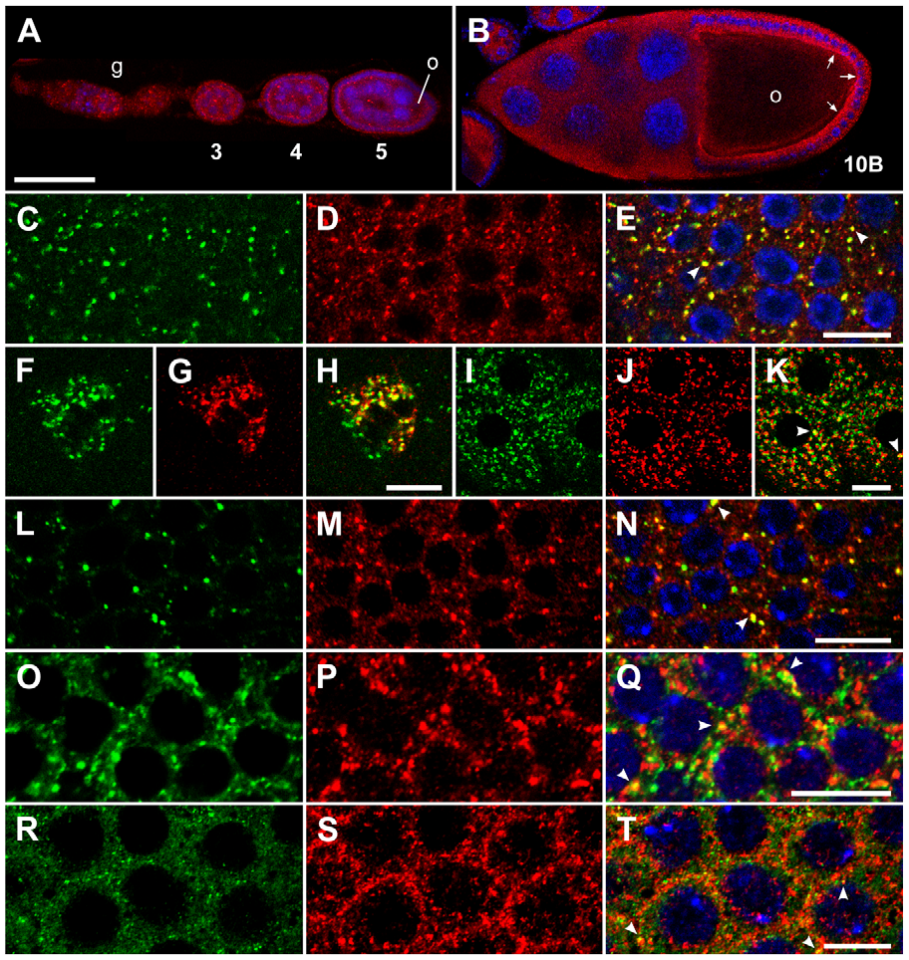
Liquid facets-Related is found throughout the ovariole and colocalizes with Golgi, clathrin and some endosome markers. (A,B) LqfR (red) is expressed in punctae in the germarium (g), in the oocyte (o), nurse cells, and in the surrounding follicle cells during oogenesis in wild type ovaries. LqfR is enriched apically (arrows) in follicle cells beginning around mid-oogenesis as is evident at stage 10B, but is much weaker in the oocyte than in surrounding tissue at this stage. Anterior is to the left and stages are indicated. (C–T) Colocalization of LqfR (red) with various markers (green). (C–E) TGN-localized anti-syntaxin16 [26] in follicle cells from a stage 9 egg chamber. medial-Golgi-localized anti-p120 [45] in migrating border cells (F–H) and in follicle cells from a stage 14 egg chamber (I–K). (L–N) Clathrin light chain-GFP [46], and (O–Q) the early endosome marker Rab5-GFP [47] in follicle cells of stage 9 egg chambers. (R–T) MVP marker anti-Hrs [48] in follicle cells from a stage 10B egg chamber. Some punctae of colocalization are indicated by arrowheads (E,K,N,Q,T). Egg chambers in (C–K, R–T) are from females of genotype *hsFLP/+* ; *FRT-82B lqfR^D66^/FRT-82B ovoD1-18*. For (L–Q), expression of the GFP-fusion proteins is driven by TubulinP-Gal4 [49] in a wild type background. Panels on the right (E,H,K,N,Q,T) are merges of the adjacent left two panels and also show To-Pro-3 stained nuclei in blue (except E and H). All images are single confocal slices. Bar, 50 µm (A,B) and 10 µm (C–T).

Vertebrate CLINT1 is expressed in all tissues [6], [7] consistent with its role in TGN-endosome trafficking, a process that should be present in all cell types and tissues. Data from the Berkeley *Drosophila* Genome Project in situ hybridization database [25] suggest that *Drosophila* LqfR is expressed primarily in the developing gut during embryogenesis. To further determine where LqfR is expressed, we performed immunofluorescence histochemistry on a variety of tissues. We found LqfR was in all tissues examined, including imaginal discs, salivary glands (data not shown), and ovaries (Fig. 2). Each ovary contains approximately 16 ovarioles, each of which contains about seven egg chambers at various stages of development [18]. As the egg chambers become more developed, they move posteriorly from the germarium (the location of stem cells for both germline- and somatic-derived cells of the egg chambers) along the ovariole toward the oviduct. Fully-formed (stage 14) egg chambers are found at the posterior-most part of the ovariole ready to be laid. In the ovary, LqfR is found in punctae within the germarium (Fig. 2A), as well as in follicle cells (somatic cells which surround the germline cells), the oocyte, and the 15 germline-derived nurse cells of egg chambers from early oogenesis (Fig. 2A,B). By stage 9, when most of the nurse cell-associated follicle cells migrate to cover the rapidly enlarging oocyte, LqfR becomes highly expressed in the apical cytoplasm of the follicle cells associated with the oocyte. Expression in the follicle cells persists through stage 14 (data not shown). Staining in the oocyte proper is comparatively weak at later stages of oogenesis (e.g. Fig. 2B).

### liquid facets-Related colocalizes with Golgi and early endosome markers

To determine the identity of the LqfR positive punctae, we performed colocalization of LqfR with a variety of markers in wildtype ovaries. LqfR colocalized strongly with syntaxin 16 (Fig. 2C–E) and colocalized partially with the p120 Golgi protein (Fig. 2F–K). Syntaxin 16 and p120 have been shown to have similar but not completely overlapping distributions [26] within the Golgi, with syntaxin 16 occupying a later Golgi compartment. Additionally, syntaxin 16 seems to function in endosome to TGN transport [27]. Significant colocalization was also seen with clathrin light chain-GFP (Fig. 2L–N) and the early endosome marker Rab5-GFP (Fig. 2O–Q). Only some colocalization was seen with the MVB marker Hrs (Fig. 2R–T) and the recycling endosome marker Rab11 (Fig. S2J–L), whereas no significant colocalization was observed with KDEL (endoplasmic reticulum), Syntaxin1 (exocytic vesicles), and Rab4 (tubulo-vesicular recycling endosome) (Fig. S2D-I). These data support a TGN and early endosome localization for LqfR, and are consistent with a role for LqfR in TGN-endosome trafficking in *Drosophila*.

### Hypomorphic *lqfR* mutants are female sterile

**Figure 3:**
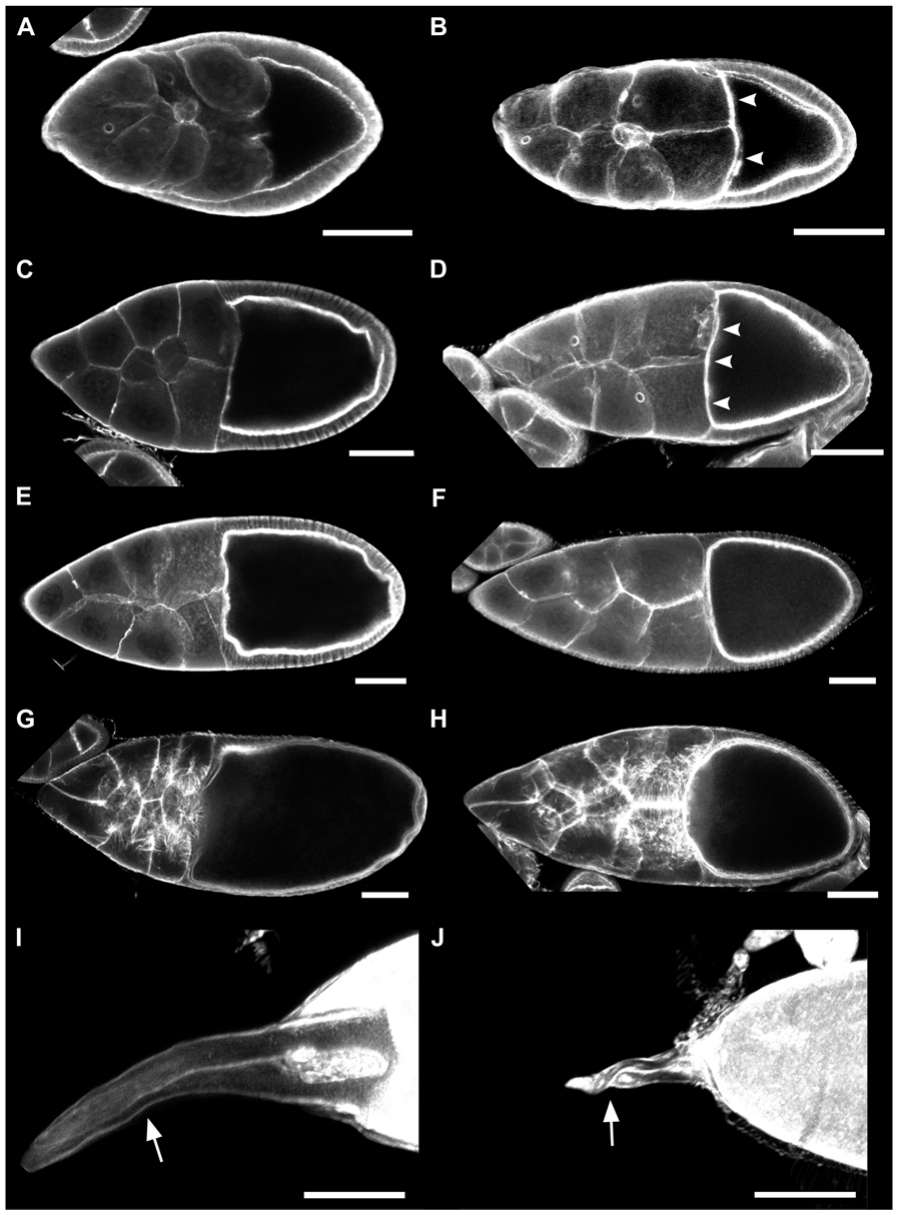
Developing egg chambers in *lqfR* mutants exhibit several morphological defects. Stage-matched TRITC-phalloidin stained egg chambers from wild type (A,C,E,G,I) and *lqfR^D66^* (B,D,F,H,J) ovaries. (A,B) Mid-stage 9; (C,D), stage 10A; (E,F) stage 10B; (G, H) stage 11 (H is slightly earlier in stage 11 than G, but it exhibits the cuboidal follicular epithelium and actin cytoskeleton of stage 11); (I,J) stage 14, anterior end only. Arrowheads show increased anterior actin in stage 9 and 10A (B,D) *lqfR* mutant egg chambers. Dorsal appendages (arrows). Anterior is at left in all panels. A–H are single confocal slices. I and J are z-stack confocal projections. Bars, 50 µm.

To determine the function of LqfR in *Drosophila*, we generated a deletion mutant by imprecise excision of a P-element found in the 5′ end of the *lqfR* genomic region. The resulting mutant, *lqfR^D66^* (Fig. 1A), contained a 1,102 bp deletion from –234 to +868 (relative to the start-ATG) and leaving behind 25 bp of P-element. This deletion removed most of the first exon, including the start-ATG, as well as most of the first intron. Although viable, fewer homozygous mutant adults eclose than would be expected by Mendelian ratios. *lqfR^D66^* appears to represent a strong hypomorphic mutation. Of note, homozygous *lqfR^D66^* flies exhibited several partially-penetrant phenotypes, most notably rough eyes in about 25% of adults (Fig. S3) and extra macrochaetae, particularly the anterior scutelars, in about 50% of adults. Melanotic inclusions were also frequently observed in the abdomens of adults. Most notably, all of the female *lqfR^D66^* mutants were sterile. Wild-type females fed an excess of yeast produce well in excess of 30 oocytes per day under optimal temperature and humidity conditions [28]. In contrast, homozygous *lqfR^D66^* females laid essentially no oocytes and the very few that were laid (∼1 per female per day) were flaccid with poorly formed dorsal appendages that failed to develop (data not shown and see Fig. 3J).

By western blot (Fig. 1E) and immunostaining (Fig. S4), *lqfR^D66^* homozygotes did not have detectable levels of protein. However, since the *lqfR^D66^* deletion does not remove the transcription start site, it is likely that *lqfR^D66^* is not a null mutation. Consistent with this observation, deletions that remove the entire *lqfR* gene are homozygous lethal at larval stages [14]. To test this, flies transheterozygous for *lqfR^D66^* were crossed to *Df(3R)Exel-6191*, a local deficiency that removes *lqfR*. While these flies were still viable and female sterile they did exhibit enhanced eye roughness (data not shown), suggesting that *lqfR^D66^* is a hypomorph.

To determine when the *lqfR* mutants start to show defects in oogenesis, we examined ovaries dissected from *lqfR^D66^* and virgin wild-type adults. After being raised on well-yeasted food for several days, ovaries from mutants were larger than those from wild-type females (data not shown), demonstrating that oogenesis was able to progress quite far and that there was an accumulation of late stage egg chambers. The larger size of ovarioles from mutants greater than four days post-eclosion resulted from the presence of two or three stage 14 oocytes, compared with only one in wild-type or heterozygous *lqfR^D66^* females (data not shown).

Closer examination of oogenesis in the mutants revealed that beginning at about the end of stage 9, when most of the follicle cells covering the nurse cells have migrated posteriorly to cover the oocyte, the egg chambers appeared elongated with the oocyte occupying less of the egg chamber than normal (Fig. 3A–H). However, migration of the border cells, a subset of approximately 7–8 anterior follicle cells that migrate to the anterior pole of the oocyte through the nurse cells, appeared normal (Fig. 3B). As vitellogenesis progresses, the egg chamber on the whole greatly increases in size, with the oocyte becoming very prominent. Normally, the oocyte should occupy approximately half the egg chamber by stage 10A [28], but the *lqfR^D66^* oocytes occupied only about a third. Stage 9 and 10A egg chambers also showed premature phalloidin staining (Figures 3, B and D) and spectrin staining (Fig. S5) at the anterior end of the oocytes. The amount of this was variable, but always appeared to be increased compared with wild-type egg chambers of the same stage. The nurse cell compartment was enlarged in late oogenesis (Fig. 3H and data not shown), which is suggestive of a defect in nurse-cell dumping, the process by which proteins and RNA required for embryogenesis are deposited into the oocyte. However, this dumping phenotype appears to be overcome, as stage 14 oocytes were of the correct size. Consistent with what was seen in the few eggs that were laid, the stage 14 oocytes showed collapsed dorsal appendages (Fig. 3I,J).

### liquid facets-Related is required in the follicle cells for oogenesis

**Figure 4:**
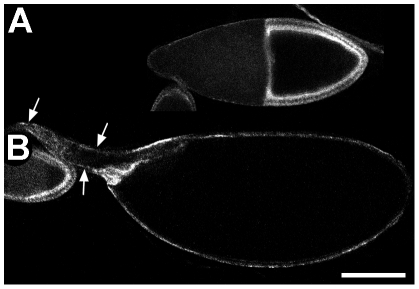
Liquid facets-related is required in the follicle cells for normal oogenesis. (A) Stage 10B and (B) stage 14 germline clone egg chambers stained with anti-LqfR H7666. Arrows show the dorsal appendages. Anterior is left. Dorsal is up in (B). Images are single confocal slices. Ovarioles are from flies with genotype *w; pr pwn hsFLP/+; FRT-82B ovoD1-18/FRT-82B lqfR^D66^*. Bar, 100 µm.

The complex phenotype observed in the egg chambers suggest that LqfR has pleiotropic effects. To address where LqfR is required in the egg chamber for oogenesis, we generated germline clones where ovarioles were null for *lqfR* in the germline, but wild-type (or heterozygous) in the follicle cells [29]. Surprisingly, strong reduction of LqfR function in the germline gave rise to fertile females with normal egg chambers (Fig. 4, Fig. S6). All such ovarioles examined had normal oocyte size relative to the length of the egg chamber from stage 9 through 11 (compare Fig. 4A with Fig. 3E,F). We also observed a normal distribution of actin from stage 10B (Fig.S4), although earlier changes in actin distribution cannot be ruled out. Finally, the dorsal appendages were restored and had a normal shape and size at stage 14 (compare Fig. 4B with Fig. 3I,J). The resulting oocytes were able to be fertilized and develop normally. This suggests that in the ovary, all aspects of the *lqfR^D66^* phenotype are due to a strong reduction of LqfR in the somatic follicle cells.

### *lqfR^D66^* clones have decreased follicle cell size

**Figure 5:**
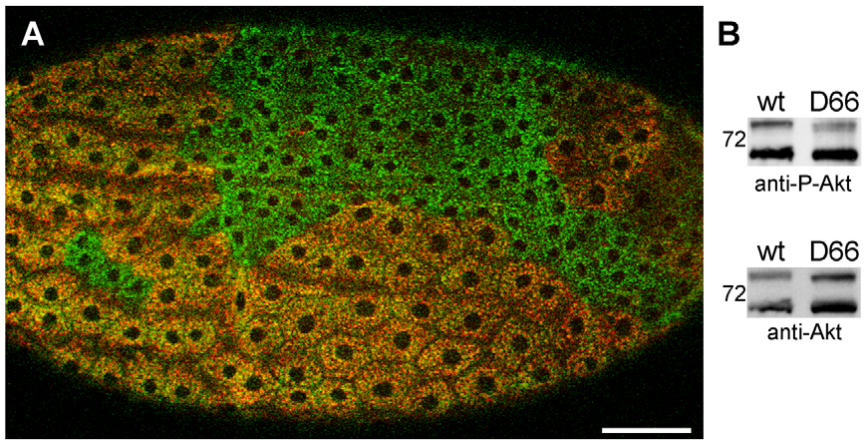
Liquid facets-Related mutants display a cell growth phenotype. (A) Follicle cells of an early stage 14 egg chamber stained with anti-p120 (green) and anti-LqfR (red) showing two *lqfR^D66^* clones. Image is a confocal z-stack projection. Anterior is to the left, dorsal is up. Bar, 50 µm. (B) Western blot of lysates from wild type and *lqfR^D66^* adult females. The blot was first probed with anti-phospho-Akt (top), then stripped and reprobed with anti-Akt (bottom). Similar results were obtained when blots were probed first with anit-Akt and then anti-phospho-Akt1 (data not shown). M*_r_* of the 72 kDa protein marker is shown.

To further define the role of LqfR in somatic follicle cells, we looked at ovarioles that contained *lqfR^D66^* clones in the follicular epithelium. We found that egg chambers from stage 9 onward that contained follicle cell clones had a defect in cell size (Fig. 5A). Specifically, the *lqfR^D66^* follicle cells were much smaller than were the adjacent wild-type cells. This size defect was dependent only on the reduction of LqfR in the follicle cells, as it was observed in ovarioles with both germline and follicle cell clones as well as in those with only follicle cell clones.

The serine/threonine kinase Akt1 acts downstream of the insulin receptor (InR) to regulate cell growth. When ligand binds InR, a cascade of events leads to the recruitment of Akt1 to the plasma membrane and its activation by phosphorylation. Activated Akt1 can then phosphorylate one of several targets, such as the forkhead transcription factor FOXO [30] and glycogen synthetase kinase 3. Signaling through Akt1 is important during *Drosophila* development. It can prevent apoptosis during embryogenesis [31], promote proper tissue morphogenesis, such as in the trachea [32], and allow normal cell growth in a variety of tissues, such as eye and wing imaginal discs [31], [33]. It is this latter role of Akt1 that has also recently been shown to regulate cell size within the follicle cells [34].

To see if the reduced cell size seen in *lqfR^D66^* clones was due to defects in Akt1 signaling, levels of phosphorylated Akt1 were compared to total Akt1 on immunoblots of lysates from adult females (Fig. 5B). When levels of total Akt1 were normalized, lysates from *lqfR^D66^* flies showed a reduction in activated, phosphorylated Akt1 to 44±1% of precise excision flies (Fig. 5C), consistent with reduced Akt1 signalling contributing to the cell size defect.

## Discussion

We have presented analysis of a gene encoding the clathrin adaptor CLINT1 in a multicellular organism. We find that a strong hypomophic mutant of *Drosophila CLINT1* (liquid facets-Related), *lqfR^D66^*, displays defects in the development of the eye and macrochaetae. These phenotypes are highly variable and are dependent on genetic background. In contrast, *lqfR^D66^* homozygous mutants are 100% female sterile. They have defects in oogenesis in the eggshell and dorsal appendages, a cell-non-autonomous defect in nurse cell dumping, and impaired cell growth of the follicle cells. Our results further show that all of these phenotypes are due to a requirement for LqfR in somatic follicle cells during oogenesis.

The *lqfR* gene structure is somewhat different than what is seen in other species. The final 924 aa of the larger product, which is encoded by exon 6, is homologous to the *S. cerevisiae* telomere length regulating protein Tel2p [35] and the related *Caenorhabditis elegans* CLK-2, which affects lifespan and may also play a role in regulating telomere length [28], [36]. While a similar genetic structure of *lqfR* with the putative telomere binding exon is found in other *Drosophilids* [20], the *CLINT1* and *Tel2p* homologs are found on different parts of the genome in *S. cerevisiae*, *C. elegans* and in vertebrates. The nature of this protein is unclear. It is predicted to share the N-terminal 492 aa with the shorter LqfR protein and was recognized by our anti-LqfR antibodies. By immunofluorescence we did not observe significant nuclear staining as might be expected with a telomere binding protein. However, a functional GFP fusion of *C. elegans* CLK-2 has a cytoplasmic localization [36]. Determining the function and significance of this larger product will require further study.

Our results with respect to the shorter *lqfR* product, which is predicted to be like vertebrate *CLINT1*, are consistent with its role in TGN–endosome trafficking. It has the expected domain structure to allow it to interact with other components of the TGN–endosome machinery and GST-LqfR did indeed bind to clathrin, γ-adaptin and GGA. Although our antibodies recognized both the short and long products, LqfR was ubiquitously expressed throughout development and co-localized with appropriate TGN and endosome markers.

The *lqfR^D66^* mutant, which behaves genetically as a strong hypomorph and affects both products, displayed a wide variety of phenotypes which together suggest that LqfR has pleiotropic effects. The mutants were not lethal, although homozygotes were produced in fewer numbers than would be predicted by Mendelian ratios. During our studies a null mutant of *lqfR* was characterized [14] which was homozygous lethal as third instar larvae, further supporting our *lqfR^D66^* mutant as a strong hypomorph. Generation of a strong hypomorph that can produce homozygous adults has allowed us to characterise the essential role of *lqfR* during oogenesis.

Since LqfR likely functions in clathrin-dependent TGN-endosome trafficking, the fact that a severe hypomorphic mutation is still viable suggests several mutually compatible possibilities. First, the reduction of the amount of protein produced in *lqfR^D66^* homozygotes is able to maintain levels of trafficking at levels sufficient to prevent cell death. Second, another protein or proteins could be functionally redundant with LqfR. Third, proteins dependent upon LqfR for trafficking may reach their appropriate destination by way of a different pathway. This could either be directly, such as via the AP-3 pathway, or indirectly perhaps via the secretory pathway to the plasma membrane and then to the endosome and lysosome via endocytosis.

Trafficking of lysosomal proteins via the cell surface may explain some of the observed phenotypes. This shunting to the plasma membrane occurs in *S. cerevisiae* missing both homologs of LqfR. When *ent3*Δ*ent5*Δ yeast are grown on plates containing milk, the mistrafficking of a portion of vacuolar enzymes causes a breakdown of milk proteins surrounding the yeast cells [11], [37]. Similarly, in vertebrate cell culture either knockdown by RNAi or overexpression of CLINT1 leads to a large increase in the amount of the lysosomal protein cathepsin D at the plasma membrane [5], [7].

If in the *lqfR^D66^* mutants lysosomal proteases were at the cell surface or secreted in small amounts due to mistrafficking it is possible that they could disrupt the chorionic structure. Mutants that exhibit similar defects to *lqfR^D66^* in the morphology of the dorsal appendages show defects in the ultrastructural organization of the eggshell [38]. Two of the mutants described in that study, *humpty dumpty* and *Origin recognition complex subunit 2*, disrupt genes encoding nuclear proteins that promote gene amplification of the chorion genes [39], [40]. The products of the chorion genes are secreted by the follicle cells to create the eggshell, including the dorsal appendages. A defect in the chorion is consistent with both the collapsed dorsal appendages seen in the stage 14 egg chambers and the flaccid oocytes that are laid.

There were several phenotypes in the nurse cells and oocytes of *lqfR^D66^* mutant ovaries that were absent from the germline clones, suggesting a role for LqfR in signaling from the follicle cells to the nurse cells and oocyte. We observed a change in the anterior actin cytoskeleton of the developing oocyte, a decrease in the relative size of the oocyte in the egg chamber, and an apparent delay in nurse cell dumping in stage 11 egg chambers (see Fig. 3). The presentation of these phenotypes is unlike what is seen in other mutants. It is possible that these could be a result of interactions between LqfR and the cytoskeleton, which is known to be important for nurse cell dumping. LqfR interacts with Cheerio, the *Drosophila* homolog of the actin binding protein filamin, in yeast two-hybrid screens [41]. Cheerio is required for proper formation of the ring canals interconnecting the nurse cells and oocyte [42], [43] and maintenance of these structures is critical for nurse cell dumping. Although the ring canals appeared normal in *lqfR* mutants (P.A.L., unpublished observations), there could be subtle defects that slow dumping. Another possibility is that LqfR is directly involved in nurse cell dumping via interactions with microtubules. The ENTH domain of rat epsins 1 and 2 can bind to microtubules [44] by way of a different part of the ENTH domain than binds phospholipids. If the ENTH domain of *Drosophila* liquid facets-Related were also able to bind microtubules LqfR might have a direct role in dumping. Arguing against the actin and microtubule binding is the germline clone experiment, which showed that LqfR is definitively required only in the follicle cells to rescue all of the observed phenotypes. This suggests that the dumping and actin phenotypes are due to impaired signaling from the follicle cells to the germline, rather than due to LqfR-cytoskeleton interactions in the germline.

The regulation of follicle cell size is known to require proper Akt1 signaling [34], and *lqfR* mutants have defects in follicle cell growth. In *lqfR* mutants, the levels of activated Akt1 were decreased leading to a decrease in Akt1 signaling and a subsequent decrease in cell size. At the same time, total Akt1 levels were elevated. The increased amount of total Akt1 could be due to an autoregulatory feedback loop. Together, these data suggest that the effect of LqfR in Akt1 signaling is upstream of Akt1 activation. However, the effect is much milder than is seen in *Akt1* hypomorphs, which are lethal except in clones.

How Akt1 signaling could be affected in the *lqfR* mutants remains unclear. Members of the pathway are either integral plasma membrane proteins, or cytosolic plasma membrane associated proteins. Neither of these classes of protein would be expected to require LqfR for their localization. Cytosolic Akt1 requires recruitment to the plasma membrane by the local production of PIP_3_ before it can be activated. An attractive model would be that PIP_3_ precursor phospholipids could be sequestered away from the cell surface in the mutant. However, endogenous expression levels of *lqfR* lacking the ENTH domain can rescue all apparent functions in an *lqfR* null mutant [14] suggesting that PIP binding by the ENTH domain is nonessential in *Drosophila*. Another possibility is the mistrafficking hypothesis raised earlier with respect to dorsal appendage formation. Akt1 signaling is instigated by insulin-like ligand binding to the InR at the plasma membrane. It is possible that in the mutant some quantity of lysosomal proteases are mistrafficked to the cell surface where they are then able to degrade some portion of either the ligand or the receptor (or both) leading to the observed decrease in Akt1 activation. Clearly this unexpected result opens up intriguing avenues for further research.

## Supporting Information

Figure S1 Alignment of *Drosophila* (Dros) and human (KIAA0171) CLINT1 proteins. Identical residues are in dark grey, and conservative substitutions are in light grey. Residue numbers are indicated at right. Residues of the ENTH domain (large box) predicted to determine specificity of phosphoinositide binding are indicated by asterisks [6], [7]. Type 2 clathrin boxes in human CLINT1 are underlined. Potential low affinity clathrin interaction sequences DLL and DLF are double underlined. γ-adaptin ear binding sequences are boxed. Alignment performed using ClustalW [50].
(TIF)

Figure S2 LqfR partially colocalizes with Rab11, but not KDEL, Syntaxin1 or Rab4. Immunostaining of follicle cells from stage 14 egg chambers with anti-LqfR (red) and other markers (green). Markers are: (A) anti-KDEL (localized to ER); (D) anti-Syntaxin1 (exocytic vesicles); (G) Rab4-RFP (tubule-vesicular recycling endosome); and (J) Rab11-GFP (rapid recycling endosome). Panels on the right (C, F, I, L) are merges of the adjacent two left panels. Tissue in (A–F) is from wild type flies. Cells in (G–I) are from flies expressing Rab4-RFP (here false coloured green) and in (J–L) from flies expressing Rab11-GFP driven by Tubulin-GAL4. Bar, 10 µm.
(TIF)

Figure S3 *lqfR^D66^* mutants have a partially penetrant rough eye phenotype. Scanning electron micrographs of eyes from (A, C) wild-type and (B, D) homozygous *lqfR^D66^* flies. Abnormally shaped ommatidia (asterisks), doubled bristles (arrows) and mis-spaced bristles (arrowheads), as well as bristles lost during sample preparation (plus signs) are indicated. Adult heads were cut in half and fixed in 4% paraformaldehyde, 2% glutaraldehyde, 0.1 M sodium phosphate, pH 7 overnight at 4°C. Following three rinses in 0.1 M PBS and dehydration in an ethanol series, samples were critical point dried and then coated with gold. Images were acquired with a Philips FEI XL30 environmental scanning electron microscope. Bar (A, B) 100 µm, (C, D) 20 µm.
(TIF)

Figure S4 Egg chambers from homozygous *lqfR^D66^* females have essentially no anti-LqfR immunoreactivity. Ovarioles from ∼2 d post-eclosion wild type (A,C) or *lqfR^D66^* (B,D) females were stained in the same dish together with anti-LqfR H7666 (green). The single confocal images were first acquired (A,B) with the settings adjusted to show staining in wild type tissue. The laser power was increased 5× to detect a similar level of fluorescence from the *lqfR^D66^* tissue, and the same ovarioles were re-imaged (C,D). Nuclei were counter-stained with To-Pro-3 (blue). The contrast in the green channel was not altered from the original image. Bar, 50 µm.
(TIF)

Figure S5 Further staining of actin cytoskeleton associated protein, spectrin, confirms *lqfR* mutants exhibit increased actin in 10A as shown in Figure 3. Stage-matched anti-spectrin (DSHB, 3A9) stained egg chambers from *lqfRD66* (A, C) and wildtype (D, F) ovaries. Nuclei are stained with DAPI (B, C). Increased anterior actin can be seen in *lqfR* mutant egg chambers. Anterior is at left in all panels. Bars, 100 um.
(TIF)

Figure S6 Females carrying *lqfRD66* germline clones have wildtype egg chambers. Stained egg chambers stage-matched to Figure 3 (E,F) are shown, costained for rabbit anti-LqfR H7666 (green) and mouse anti-spectrin (red; 3A9 from DSHB). The *lqfRD66* germline clones do not have LqfR staining (A) but have an actin cytoskeleton appearance (B) similar to wild type (Figure 3E). *lqfRD66* mutants show increased anterior actin and increases in the relative size of the nurse cell compartment (C–E). Stage matched, wild type egg chambers are shown for comparison (C′–E′). Anterior is at left in all panels.Bars, 100 mm.
(TIF)
