# Supplementary figures and images for: Liquid facets-Related (lqfR) Is Required for Egg Chamber Morphogenesis during Drosophila Oogenesis
Source: PLoS One. 2011 Oct 17;6(10):e25466. doi: 10.1371/journal.pone.0025466 (PMC3197181; doi:10.1371/journal.pone.0025466)

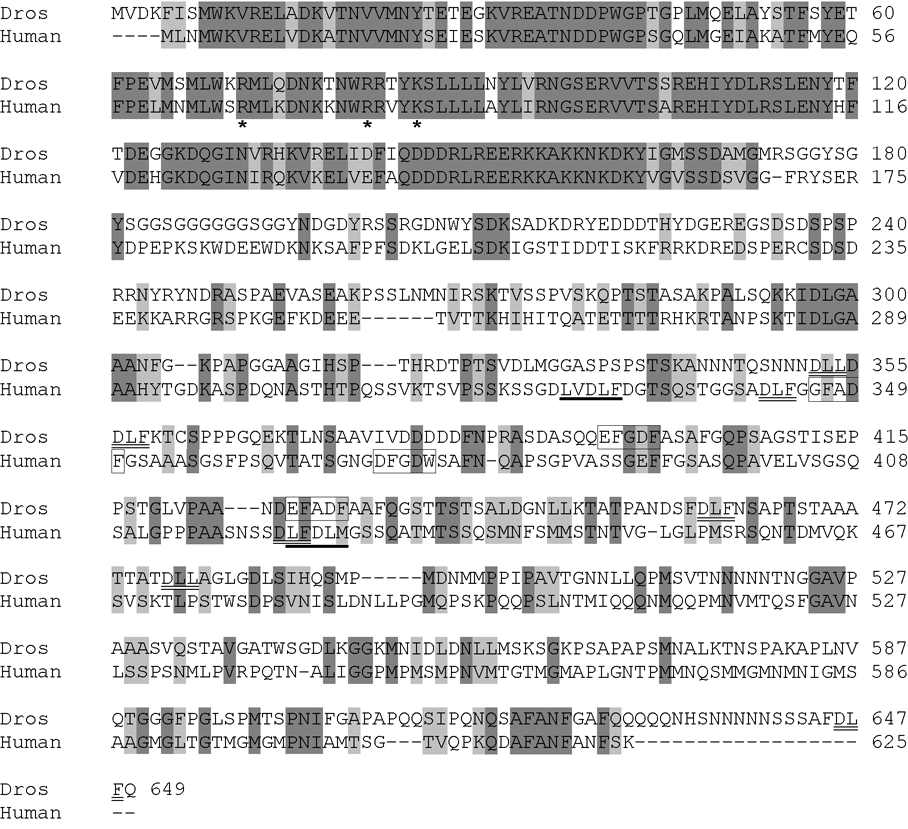

Supplement: Figure S1 — Alignment of Drosophila (Dros) and human (KIAA0171) CLINT1 proteins. Identical residues are in dark grey, and conservative substitutions are in light grey. Residue numbers are indicated at right. Residues of the ENTH domain (large box) predicted to determine specificity of phosphoinositide binding are indicated by asterisks [6], [7]. Type 2 clathrin boxes in human CLINT1 are underlined. Potential low affinity clathrin interaction sequences DLL and DLF are double underlined. γ-adaptin ear binding sequences are boxed. Alignment performed using ClustalW [50]. (TIF) [file pone.0025466.s001.tif]

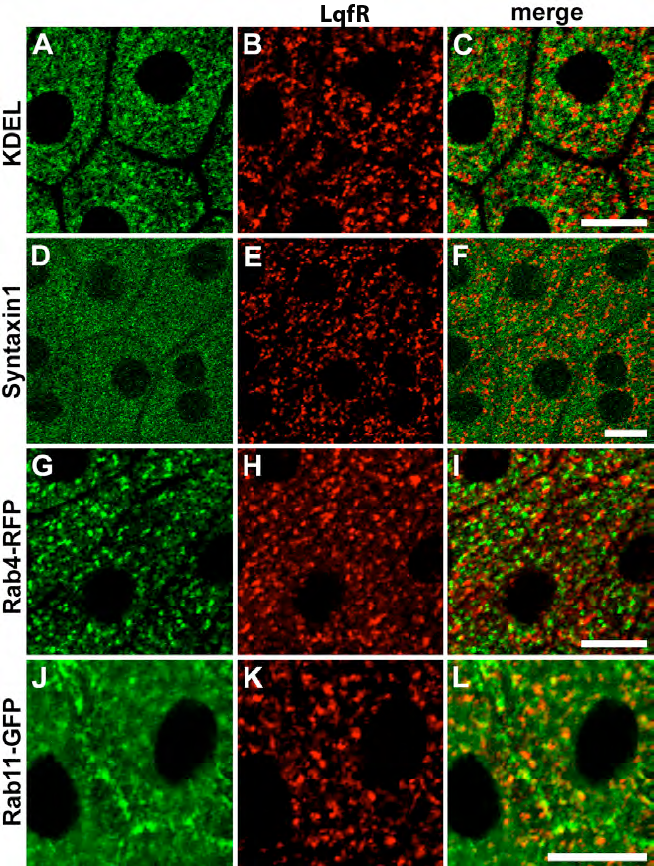

Supplement: Figure S2 — LqfR partially colocalizes with Rab11, but not KDEL, Syntaxin1 or Rab4. Immunostaining of follicle cells from stage 14 egg chambers with anti-LqfR (red) and other markers (green). Markers are: (A) anti-KDEL (localized to ER); (D) anti-Syntaxin1 (exocytic vesicles); (G) Rab4-RFP (tubule-vesicular recycling endosome); and (J) Rab11-GFP (rapid recycling endosome). Panels on the right (C, F, I, L) are merges of the adjacent two left panels. Tissue in (A–F) is from wild type flies. Cells in (G–I) are from flies expressing Rab4-RFP (here false coloured green) and in (J–L) from flies expressing Rab11-GFP driven by Tubulin-GAL4. Bar, 10 µm. (TIF) [file pone.0025466.s002.tif]

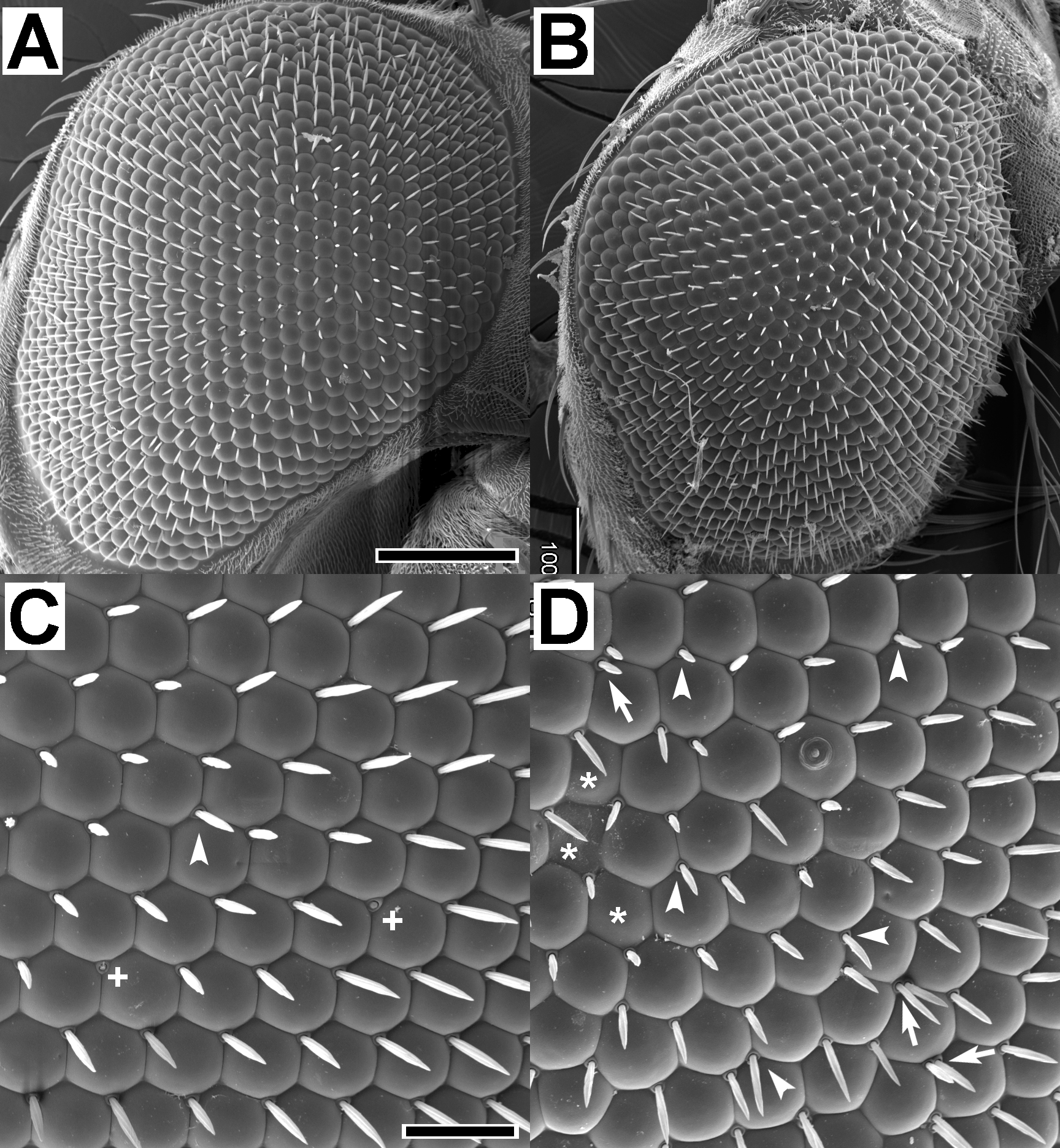

Supplement: Figure S3 — lqfRD66 mutants have a partially penetrant rough eye phenotype. Scanning electron micrographs of eyes from (A, C) wild-type and (B, D) homozygous lqfRD66 flies. Abnormally shaped ommatidia (asterisks), doubled bristles (arrows) and mis-spaced bristles (arrowheads), as well as bristles lost during sample preparation (plus signs) are indicated. Adult heads were cut in half and fixed in 4% paraformaldehyde, 2% glutaraldehyde, 0.1 M sodium phosphate, pH 7 overnight at 4°C. Following three rinses in 0.1 M PBS and dehydration in an ethanol series, samples were critical point dried and then coated with gold. Images were acquired with a Philips FEI XL30 environmental scanning electron microscope. Bar (A, B) 100 µm, (C, D) 20 µm. (TIF) [file pone.0025466.s003.tif]

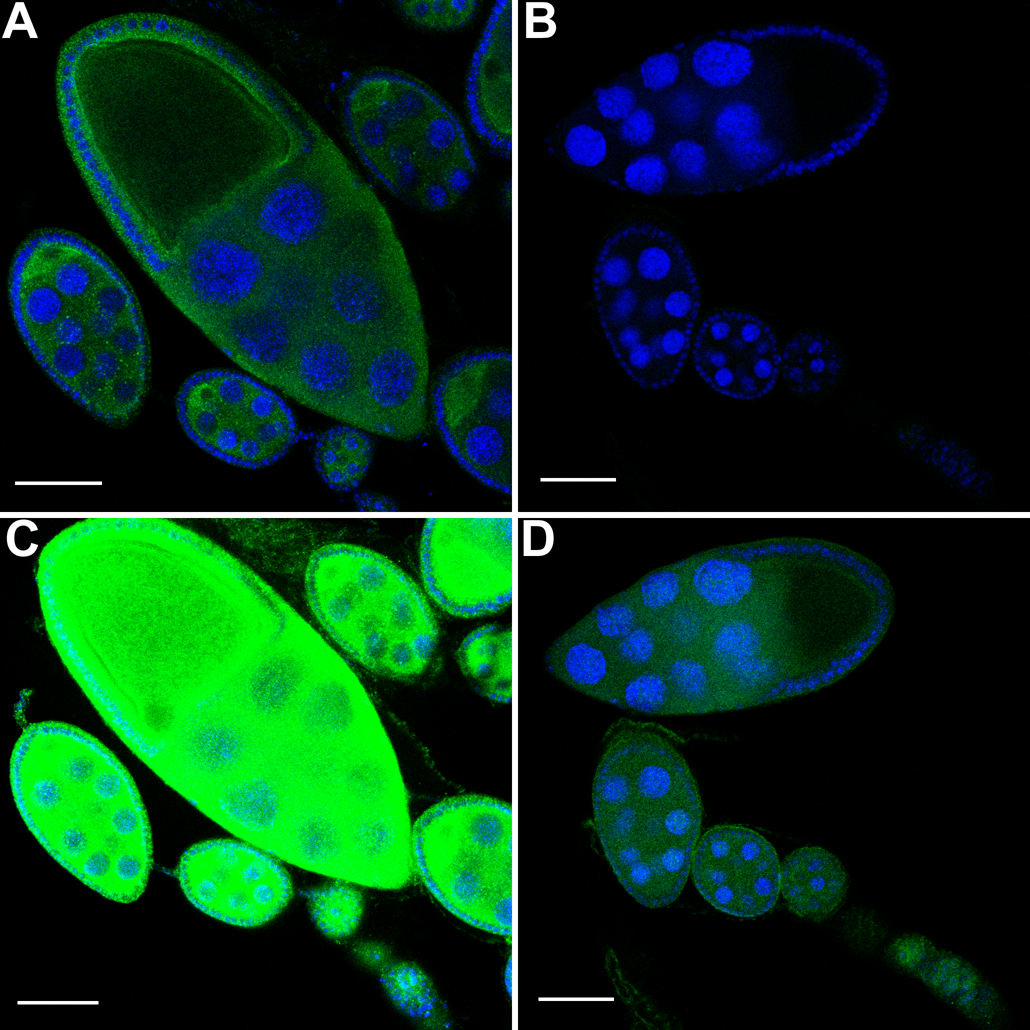

Supplement: Figure S4 — Egg chambers from homozygous lqfRD66 females have essentially no anti-LqfR immunoreactivity. Ovarioles from ∼2 d post-eclosion wild type (A,C) or lqfRD66 (B,D) females were stained in the same dish together with anti-LqfR H7666 (green). The single confocal images were first acquired (A,B) with the settings adjusted to show staining in wild type tissue. The laser power was increased 5× to detect a similar level of fluorescence from the lqfRD66 tissue, and the same ovarioles were re-imaged (C,D). Nuclei were counter-stained with To-Pro-3 (blue). The contrast in the green channel was not altered from the original image. Bar, 50 µm. (TIF) [file pone.0025466.s004.tif]

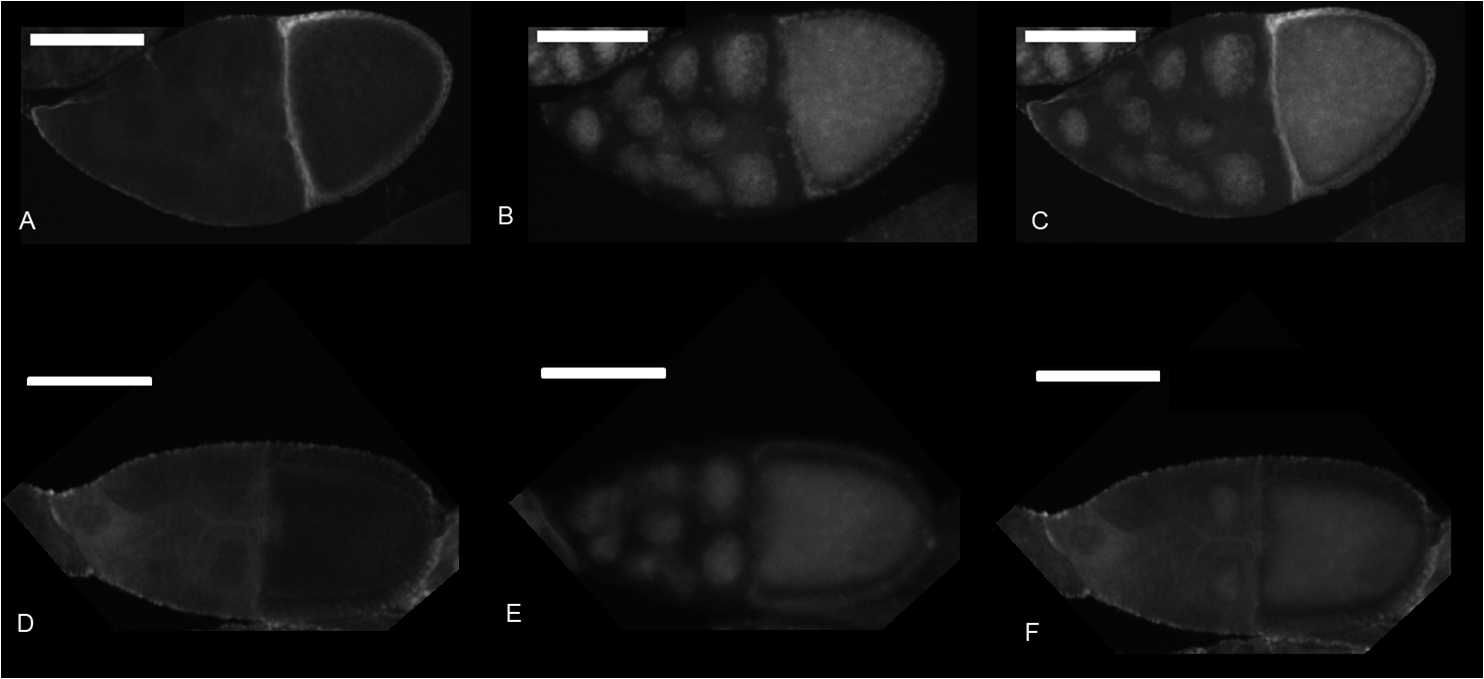

Supplement: Figure S5 — Further staining of actin cytoskeleton associated protein, spectrin, confirms lqfR mutants exhibit increased actin in 10A as shown in Figure 3. Stage-matched anti-spectrin (DSHB, 3A9) stained egg chambers from lqfRD66 (A, C) and wildtype (D, F) ovaries. Nuclei are stained with DAPI (B, C). Increased anterior actin can be seen in lqfR mutant egg chambers. Anterior is at left in all panels. Bars, 100 um. (TIF) [file pone.0025466.s005.tif]

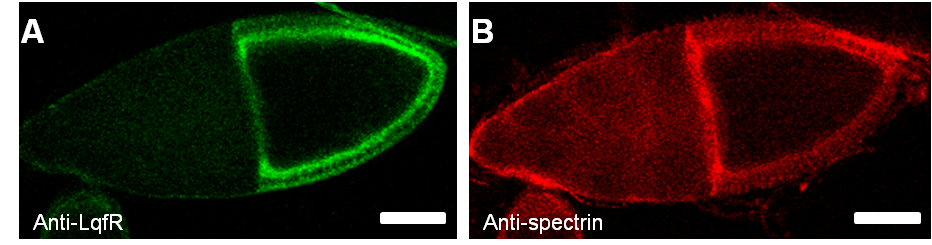

Supplement: Figure S6 — Females carrying lqfRD66 germline clones have wildtype egg chambers. Stained egg chambers stage-matched to Figure 3 (E,F) are shown, costained for rabbit anti-LqfR H7666 (green) and mouse anti-spectrin (red; 3A9 from DSHB). The lqfRD66 germline clones do not have LqfR staining (A) but have an actin cytoskeleton appearance (B) similar to wild type (Figure 3E). lqfRD66 mutants show increased anterior actin and increases in the relative size of the nurse cell compartment (C–E). Stage matched, wild type egg chambers are shown for comparison (C′–E′). Anterior is at left in all panels.Bars, 100 mm. (TIF) [file pone.0025466.s006.tif]
